# Investigation on the Modeling and Reconstruction of Head Injury Accident Using ABAQUS/Explicit

**DOI:** 10.3390/bioengineering9120723

**Published:** 2022-11-23

**Authors:** Xingqiao Deng, Zhifei Du, Huiling Feng, Shisong Wang, Heng Luo, Yucheng Liu

**Affiliations:** 1 College of Mechanical and Electrical Engineering, Chengdu University of Technology, Chengdu 610059, China; 2 Hongguang Street Health Center, Pidu District, Chengdu 610097, China; 3 Department of Mechanical Engineering, South Dakota State University, Brookings, SD 57006, USA

**Keywords:** accident reconstruction, skull fracture, head injury, damage biomechanics, head-to-ground simulation

## Abstract

A process of modeling and reconstructing human head injuries involved in traffic crashes based on ABAQUS/Explicit is presented in this paper. A high-fidelity finite element (FE) model previously developed by the authors is employed to simulate a real accident case that led to head injury. The most probable head impact position informed by CT images is used for the FE modeling and simulation since the head impact position is critical for accident reconstruction and future analysis of accidents that involve human head injuries. Critical von Mises stress on the skull surface of the head model is chosen as the evaluation criterion for the head injury and FE simulations on 60 cases with various human head—concrete ground impact conditions (impact speeds and angles) were run to obtain those stress values. The FE simulation results are compared with the CT images to determine the minimum speed that will cause skull fracture and the corresponding contact angle at that speed. Our study shows that the minimum speed that would cause skull fracture is 3.5 m/s when the contact angle between the occipital position of the injured head and the ground is about 30°. Effects of the impact speed and the contact angle on the maximum von Mises stress of the head model are revealed from the simulations. The method presented in this paper will help forensic pathologists to examine the head impact injuries and find out the real reasons that lead to those injuries.

## 1. Introduction

Traffic injuries and fatalities are becoming a global concern due to the dramatic increase in road traffic crashes, which has caused deaths, financial losses, and other unbearable losses to families and society. In 2018, the World Health Organization (WHO) reported an annual 1.35 million traffic deaths and up to 50 million traffic injuries [1]. Among all the car crash injuries, 30% of them were head injuries, including skull fracture and brain damage, and many other injuries, diseases, and sequelae such as the vegetative state, blindness, migraines, and mental illness. In-depth investigations of these biomechanical phenomena behind traumatic brain injury are indispensable for understanding the relationship between human head responses and various vehicle–head impact scenarios. Results of such investigations can also be used as critical proof for forensic analysis of craniocerebral injury in traffic crashes.

Despite tremendous efforts and resources invested on head injury study, the barriers and issues in the analysis of head injury incurred in traffic crashes still remain unsolved. Because a traffic crash is instantaneous and involves a complicated, high-rate process, a rough estimation may lead to wrong conclusions. In most cases, an accurate description of the entire process is almost impossible due to time restrictions for road closures as well as other constraints. Thus, the real cause of the traffic crash can hardly be obtained. On the other side, a thorough inspection of head injuries is essential in forensic investigations because it is critical for pathologists to understand the injury mechanism so as to determine whether such injuries are accidental or inflicted.

During the second half of the 20th century, a number of mathematical models were developed based on Newton’s principles and experimental observations for the purpose of predicting human head injury mechanisms in given situations. A comprehensive review of those models was performed by Cory et al. in 2001 [2]. However, the accuracy of those models in reproducing the traffic crashes is relatively low due to errors in the acquired experimental data. In vitro experiments directly performed on human specimens can provide correct data, but such experiments require high precision measurement equipment, which are not only very costly, but also intend to cause ethical disputes. In many experimental studies, physical or biological substitutes were tested to understand the relationship between clinical symptoms and brain injury mechanisms. However, due to the complex dynamic loading conditions that victims are subjected to, it is almost impossible to reconstruct real head injuries incurred in accidents [3]. Besides the experimental approaches, use of scalable computers provided a promising, safe, and cost-effective approach to evaluate the consequences of a given head impact by calculating the stress and strain of the brain under a high energy shock through finite element (FE) analysis and simulation [4].

As demonstrated before, due to various constraints such as the time restriction for road closures and technology limitations, the data and information collected from scenes of traffic crashes are sometimes incomplete and may even contain considerable errors. This makes it extremely difficult to conduct on-site investigations of pedestrian collision accidents or any post-accident analysis based on the data acquired from the scenes of accidents. In other words, in order to yield quality data that can be used to reveal the head’s biomechanical response during severe impacts, the scenes of traffic crashes need to be reconstructed and entire impact processes have to be reproduced. In this regard, computational modeling and simulation is the most powerful tool for this purpose.

Traffic crash research includes traffic crash analysis, reproduction, and statistical analysis [5]. The traffic accident reconstruction is a process of making inferences about the cause of the traffic crash based on the vehicle damage, personal injury situation, and various forms of traces on the scene of the traffic crash, referring to the accounts of the parties and witnesses. A correct and comprehensive analysis of a traffic crash almost equals to repeating that accident, from which much valuable information and data are revealed, which would hardly be available otherwise. Thus, the traffic accident reconstruction plays a crucial role in the passenger/pedestrian injury risk prediction. It also provides a scientific basis for the transportation department in decision making and assists the jury in deciding key issues in a traffic crash case. In short, the reproduction of traffic crashes is a key component in the traffic crash research. As verified in numerous previous studies, simulation results can provide more valuable insights about the traffic crashes and assist traffic officers in identifying responsibilities of involved parties and revealing the courses of accidents.

For example, Deng et al. investigated and assessed the dependence of dummy head injury mitigation on the side curtain airbag and occupant distance under a side impact of a Dodge Neon via full-scale FE simulations [6]. Chai et al. proposed a method for traffic crash scene reconstruction and the process reconstruction by combining digital reconstruction and computer simulation technology [7]. Berthelson et al. employed a combined FE–mathematical surrogate modeling approach to study car crash-induced neck injury trends in response to variations in traffic crash parameters at significantly-reduced number of necessary FE simulations [8]. Cui et al. developed a precise FE model of the head and neck based on human anatomical structure and used that model to study neck injuries caused by rear impact at different speeds [9]. He et al. conducted two-dimensional mesoscale FE analysis of a multi-layered brain tissue to calculate the damage-related average stress triaxiality and local maximum von Mises strain in the brain [10]. In their work, behaviors of wet and dry brain issues were described by integrating the FE model with a rate-dependent hyperelastic and internal state variable method. All these works have proved that the combination of FE modeling and simulation and the traffic crash identification technology can effectively enhance the capacity in reconstructing the impact process in traffic crashes, therefore promoting the impact head injury analysis.

Because of its advantages in modeling complicated shapes and simulating real physical processes, FE methods have been extensively used for evaluating the crashworthiness of vehicle structures [11,12,13,14], designing thin-walled energy absorbers [15,16], and investigating head injuries incurred in different collision events [17]. With a high fidelity and geometrically accurate human head model, the FE methods are considered the best tool for studying head response under controlled impact conditions [18,19]. Many high-fidelity FE human head models have been developed, and those developed models provide a scientific background for the present study. Ruan et al. [20] established a detailed FE head model consisting of 6080 nodes and 7351 elements in 1994. Zhou and coworkers improved that model to a more detailed one that consisted of 17,656 sections and 22,995 units. The enhanced model even differentiated the white and gray matter in the brain [21]. In 2001, Zhang et al. continued to revise that model by redefining the sliding interface between the skull and brain model [22]. Kleiven and Hardy developed another FE head model in 2002 and used that model to study the biomechanical response of the brain in low-severity impacts, including the relative motion between the skull and the brain [23]. Horgan and Gilchrist constructed a 3D FE representation of the human head complex for simulation the transient occurrences of simple pedestrian accidents [24].

Since 2016, more physics-based, biofidelic human head models have been developed using advanced modeling methods and novel constitutive laws. Those state-of-the-art FE head models have been used for experiment-informed simulations to faithfully reproduce the traffic crashes and human head response under impact loading. Sahoo et al. [25] enhanced an existing FE head model with composite modeling and a new constitutive law for the skull. The response of their FE head model was validated using data from 15 temporo–parietal impact experiments, conducted with postmortem human surrogates. Cotton et al. [26] proposed a new modeling methodology that addressed several limitations of conventional FE head models. They employed that methodology to develop a highly bio-fidelic FE head model from high-resolution scan data. The accuracy and feasibility of the model were successfully demonstrated against a widely used experimental benchmark in impact loading and through the investigation of a potential brain injury under blast overpressure loading. Trotta and coworkers [27] reported the development of a biofidelic FE head model originally developed from a brain trauma model established in 2003, which accurately represents the mechanical and sliding properties of the scalp.

Despite the numerous efforts in simulating the biomechanical response of the human head during human–vehicle impacts, a few studies were dedicated to understanding the head injury and damage caused by human–ground impacts. The human–ground impact is caused by falls of victims in car–pedestrian accidents, which is the top cause of traumatic brain injuries. Han et al. [28] reconstructed electric two-wheeler riders’ head-to-ground impact accidents using FE simulations. In their simulations, the victim wore a protective helmet. To the authors’ best knowledge, interactions between a bare head and the ground during the human–ground impacts have not been simulated and thoroughly studied. The goal of the present study is following the FE modeling approach to use a high-fidelity FE head model to simulate the head–ground impact involved in a real accident case, which led to significant head injury. The experiment-informed, head-to-ground simulations are supported by detailed accident data such as CT images.

In this paper, a high-fidelity FE head model as well as a modeling approach is developed for understanding the interaction between a bare head and the ground during human–ground impacts and revealing the minimum impact speed that would cause skull fracture and the corresponding contact angle. Based on the present model and the proposed modeling approach, advanced FE models can be developed to study actual head injuries including secondary injuries by taking different factors (e.g., weather, road conditions, human factors) into consideration.

## 2. Methodology

### 2.1. FE Human Head Model

**Figure 1:**
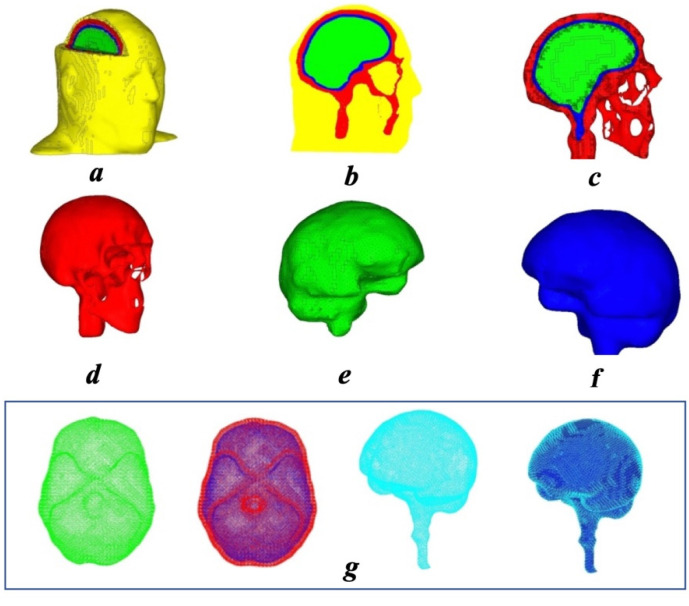
The FE head model: (**a**) isometric view of the head model with brain exposed; (**b**) medium sagittal view of the head model; (**c**) sectional view of skull and brain model; (**d**) skull and facial bones model; (**e**) brain model; (**f**) cerebrospinal fluid model; (**g**) skull–brain interface model.

The FE head model used for this study was developed and validated by one author of this paper (Deng) and a team of researchers at Mississippi State University based on a set of high-resolution computer tomography (CT) images provided by the National Library of Medicine [29]. As shown in Figure 1, in generating this high-fidelity model, the head model was first divided into four parts (skull, brain, cerebrospinal fluid, and the outer contour of the head) using image processing software ScanIP. After that, pre-processing software Hypermesh was applied to generate natural, high-quality FE meshes. These FE elements were generated so that they retained the anatomical features of the human head, scalp, skull, cerebrospinal fluid, and brain. Figure 1 shows the model of the human head and head–brain interface, which consists of 472,061 elements and 1,404,580 nodes (the skull and scalp/skin only). The element size and mesh density were carefully chosen following Liu and Glass’ method [30] to reach an optimal tradeoff between accuracy and computing speed.

Numbers of elements for each part of this head model are listed in Table 1.

### 2.2. Material Properties

To obtain accurate simulation results, properties of all materials of this head model have to be precisely defined. A number of investigators obtained the head material properties from experiments and published them in references [18,19,20,21,22,23]. Those properties are used to define the materials of our head model. For instance, the skull, meninges, and skin are modeled as elastic materials, while the cerebrospinal fluid and brain tissue are defined as viscoelastic materials. All the material properties are displayed in Table 2 and Table 3. As verified by the author [29], the present head model and adopted material properties are capable of correctly capturing the head response under different impact conditions.

### 2.3. Numerical Algorithm

The ABAQUS/Explicit Dynamic software tool is used for simulating the high-speed impact between the human head and the ground. Compared with the implicit solver, the explicit solver is more suitable for simulating dynamic processes which involve high strain rates, nonlinearity, fast changing stress and strain states, as well as large deformation and even damage of materials because it does not require an implicit tangent stiffness matrix and does not involve any iterations or convergence criteria [31]. We used the ABAQUS/Explicit solver for the present study because of its efficiency in multi-object collision simulations.

### 2.4. Head–Ground Impact Simulations

**Figure 2:**
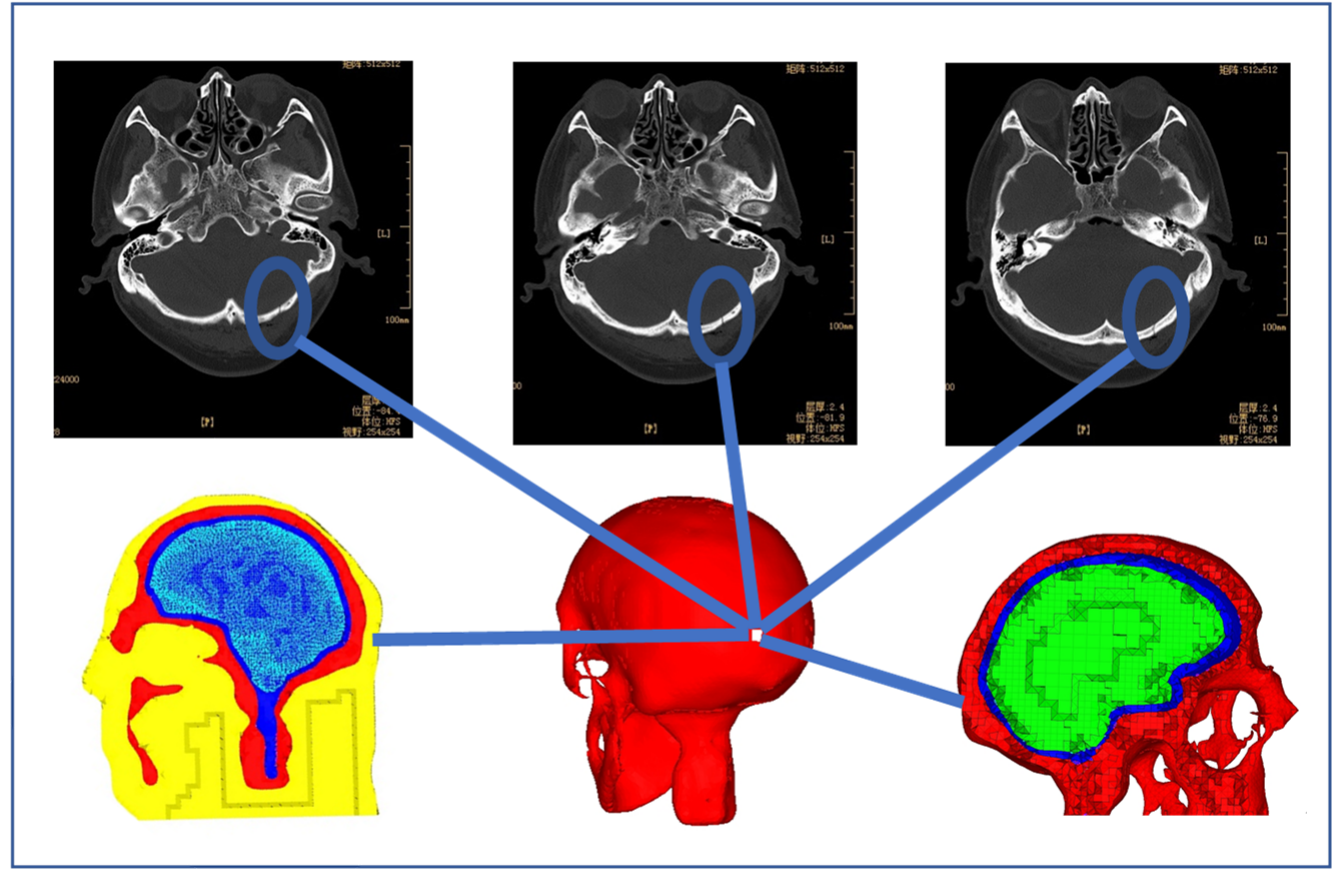
CT images showing the head injury, which includes extensive intracranial contusion and pneumatosis, hemorrhage, and occipital fracture.

**Figure 3:**
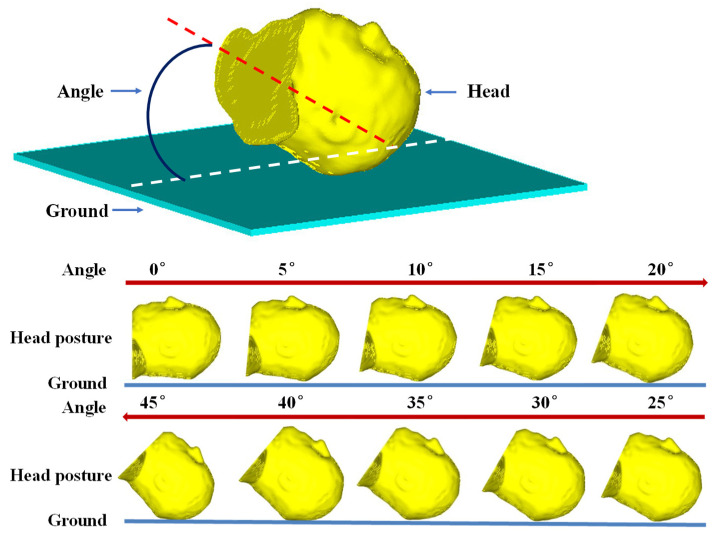
Contact between the head and the ground at different angles.

CT images displayed in Figure 2 indicate the impact position where the stress and strain of the head model were monitored. The location of the injury was identified in the CT images, which came from a real head–ground impact incurred by a pedestrian–vehicle collision. Boundary and loading conditions such as the impact speed and contact angle between the head and ground were defined for the simulation. The head posture angle was varied between 0° and 45° and the stresses around the known location of the injury were calculated. The velocity of the impact was predetermined, which was varied between 1 m/s and 3.5 m/s. In the simulations, an initial, unified velocity field was applied on all nodes of the head model and the ground was fully constrained. Figure 3 shows the contact between the head model and the ground, where the contact location was determined from the information provided in the CT images (Figure 2). A face-to-face contact type was defined at the interface between the ground and the head, where the ground was modeled as a flat surface. The simulation time step was 1× 10^−8^ s, which was small enough for dynamic analysis. After each simulation, the peak stress and stress profile at the position corresponding to the skull injury occurring in the real accident (Figure 2) were calculated and monitored.

According to Shi et al. [32], head injuries due to ground impact are not likely caused by head rotation but instead resulted from the direct head-to-ground impact. Thus, in our simulations, an initial and unified velocity field is applied on all nodes in the head model and the ground is fully constrained. The CT images shown in Figure 2 indicate where the skull fracture occurred, which is decided as the head-to-ground contact point (Figure 3). Stress and strain variations at that point and the neighboring area are monitored in our simulations.

Figure 3 shows different contact angles between the head and the ground. The contact point was determined based on the CT images (Figure 2) and the head-to-ground contact was defined as surface-to-surface contact. The time step was 1 × 10^−8^ s, which was small enough for dynamic simulations. The maximum von Mises stress at that point and the stress profile on the head model were calculated in each simulation.

### 2.5. Head Injury Threshold Criteria

In human head injury and skull fracture analysis, stress and strain data are better predictors compared with linear and angular accelerations [27]. Therefore, in such studies, the post-traumatic biomechanical response and parameters, such as intracranial pressure (ICP), von Mises stress, and strain, are used to evaluate the severity of traumatic brain injury by comparing the simulation results with the tolerance thresholds of brain tissue [33]. In this study, we chose von Mises stress to measure the severity of head injury and skull fracture based on the seminal work conducted by McElhaney et al. [34]. The threshold von Mises stress values for skull fracture were obtained by McElhaney et al. [34] and are used here as the yield limit of the skull to predict the potential fracture. Table 4 lists those values. The same set of criteria was also used by Yang [4], Trotta [27], and Kim [33] for head and brain injury analysis and prevention.

## 3. Results

### 3.1. Skull Injury under Different Impact Conditions

**Figure 4:**
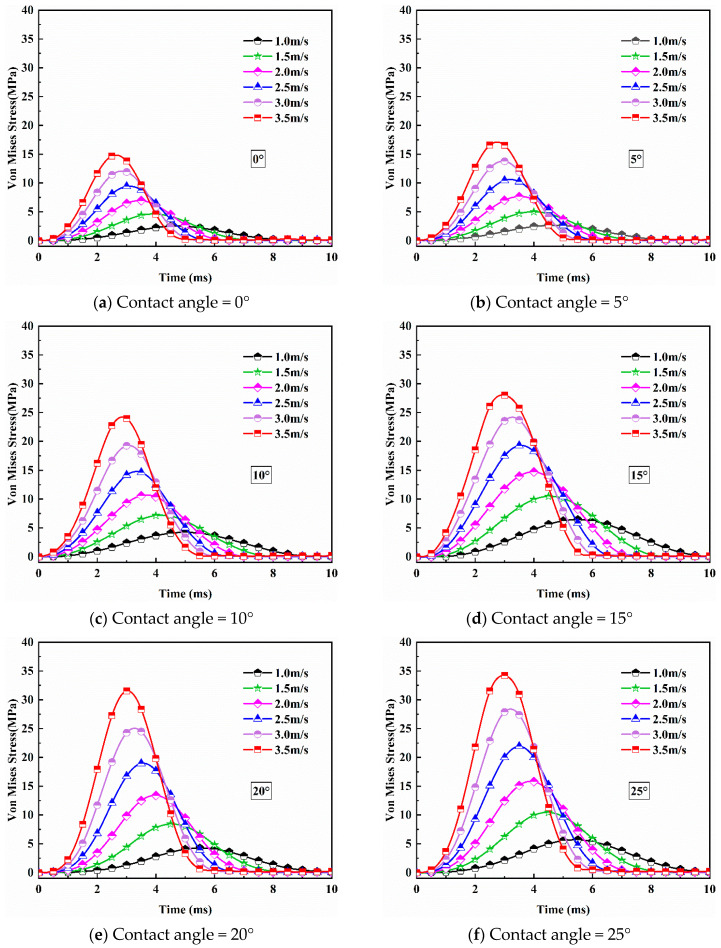

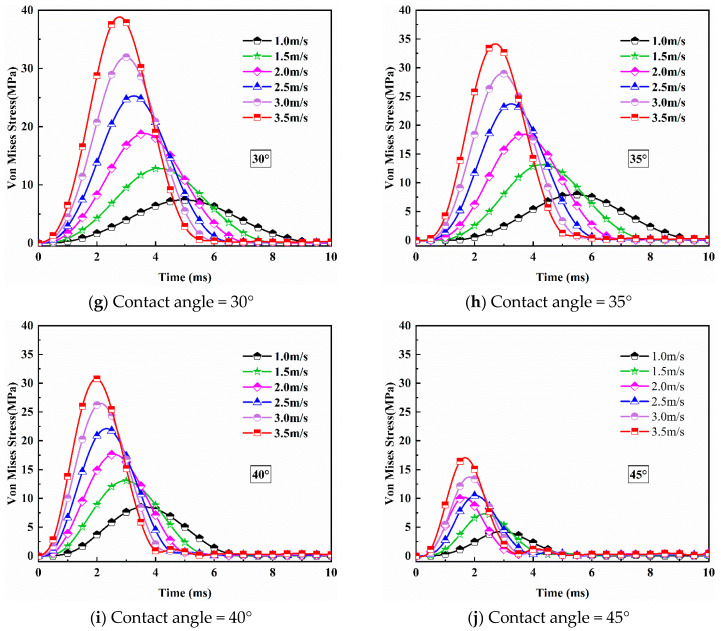
Stress curves at different impact conditions.

Figure 4 plots stress curves calculated at the position indicated in Figure 2 when the impact speeds are 1 m/s, 1.5 m/s, 2 m/s, 2.5 m/s, 3 m/s, and 3.5 m/s at different contact angles ranging from 0° to 45°. One goal of this study is to find the minimum speed and the contact angle that would cause head injury. Therefore, we start from a very low speed (1 m/s) and gradually increase the impact speed until the maximum von Mises stress on the head model first exceeds the threshold value. At each impact speed, different contact angles ranging from 0° to 45° are entered for simulations (as shown in Figure 3, the contact angle cannot exceed 45°). After each simulation, the maximum von Mises stress is calculated and compared with the threshold value. It is not until the speed reaches 3.5 m/s that the peak stress for the first time exceeds the threshold value when the contact angle is 30°. In our simulations, the increases of 0.5 m/s (impact speed) and 5° (contact angle) were selected to reduce the simulation work.

**Figure 5:**
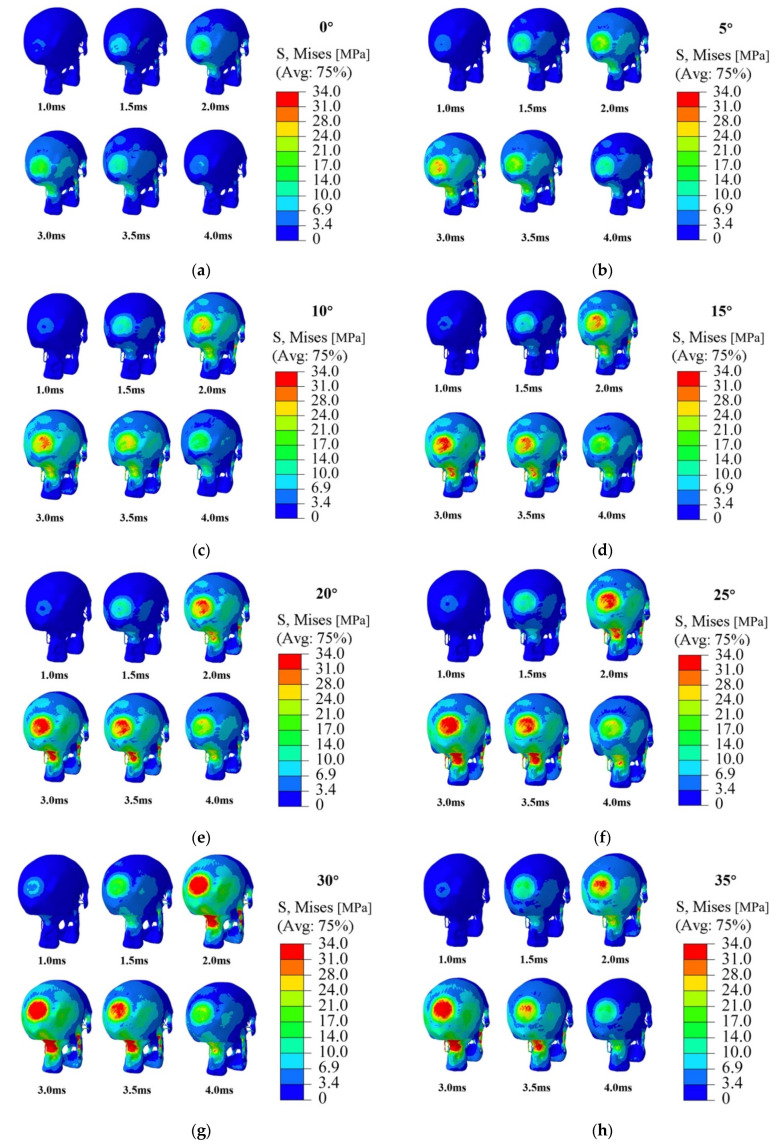

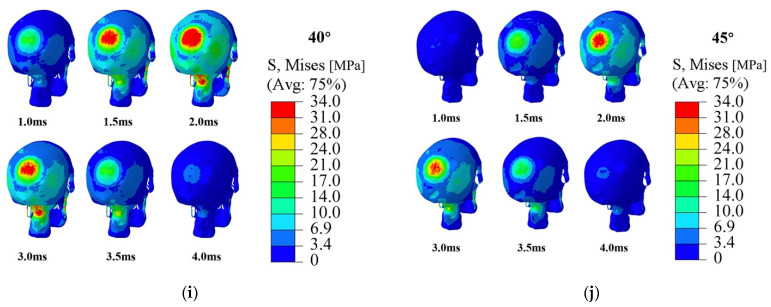
Stress nephograms generated from impact simulations at 3.5 m/s with different contact angles: (**a**) 0°, (**b**) 5°, (**c**) 10°, (**d**) 15°, (**e**) 20°, (**f**) 25°, (**g**) 30°, (**h**) 35°, (**i**) 40°, (**j**) 45°.

It can be seen from Figure 4 that during an entire impact process, the stress first increases with the increase in time and reaches the maximum value. This process represents that the human head begins to deform after touching the ground and finally reaches the maximum state of deformation. After reaching the peak stress, the stress value gradually decreases from that peak value to 0. This is a rebound process in which the head separates from the ground and the deformation of the head gradually returns to its normal state from the maximum deformation. In addition, as the impact speed increases, the magnitude of the peak stress also increases. Apparently, a high-speed impact will cause more extensive damage to the head. From the same figure, it can also be found that at the highest impact speed (3.5 m/s), the obtained peak stress value (37.84 MPa) first reaches the threshold value for skull fracture (34.47 MPa) when the contact angle is 30°. For other cases with lower speeds, the peak stress values are also achieved at the contact angle of 30° while they are lower than 34.47 MPa and will not lead to the damage of the skull. Therefore, it can be deduced that the head–ground impact will lead to the most severe injury to the brain when the head hit the ground at an angle of 30°. From Figure 5, we can see that as the impact speed increases, the stress nephogram changes significantly.

Focusing on the maximum stress and stress variation occurring at the highest impact speed of 3.5 m/s, Figure 5 displays the stress profiles calculated from the impact simulations at that speed but different contact angles at a series of times (1 ms, 1.5 ms, 2 ms, 3 ms, 3.5 ms, and 4 ms). From the images in that figure, the history and variation of maximum stress and stress distribution on the entire head model during an entire impact process are clearly shown. It can be found that during the impact, the human head is subjected to severe injury at two locations: the contact location between the head and the ground and the junction between the skull and the spine. This phenomenon becomes more obvious after 2 ms since the initial contact. In other words, besides the impact position, the junction between the skull and spine will also be severely injured during the impact process.

### 3.2. Effects of Impact Angle on Peak Stress Value of the Skull

**Figure 6:**
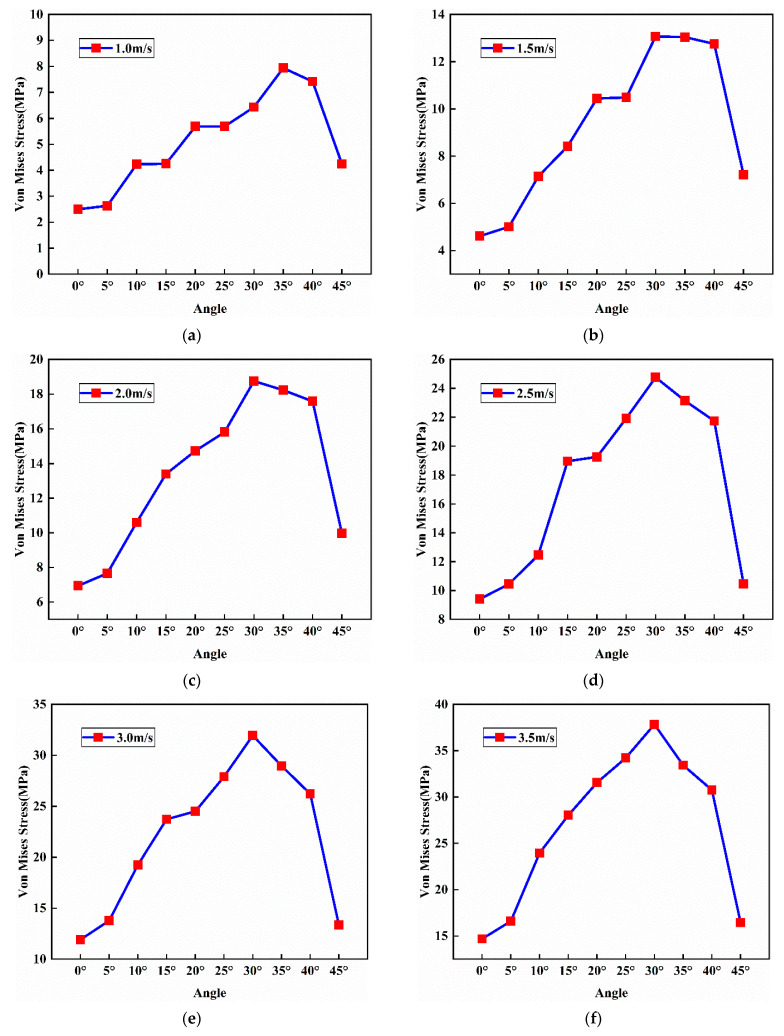
Peak stress values on the skull obtained from different impact conditions: (**a**) impact speed v = 1.0 m/s, (**b**) v = 1.5 m/s, (**c**) v = 2.0 m/s, (**d**) v = 2.5 m/s, (**e**) v = 3.0 m/s, (**f**) v = 3.5 m/s.

Figure 6 shows how different impact angles affect the peak stress on the skull. As explained before, the peak stress value occurring during the impact indicates the severity of the head injury. From the figure, it is clear that as the contact angle increases from 0° to 30°, the peak stress on the skull developed during the impact also increases regardless of the impact speed. However, when the contact angle changes from 30° to 45°, the peak stress decreases as the contact angle increases. Therefore, it can be deduced that the maximum peak stress as well as the maximum damage during the impact occurs when the contact angle is about 30°.

**Figure 7:**
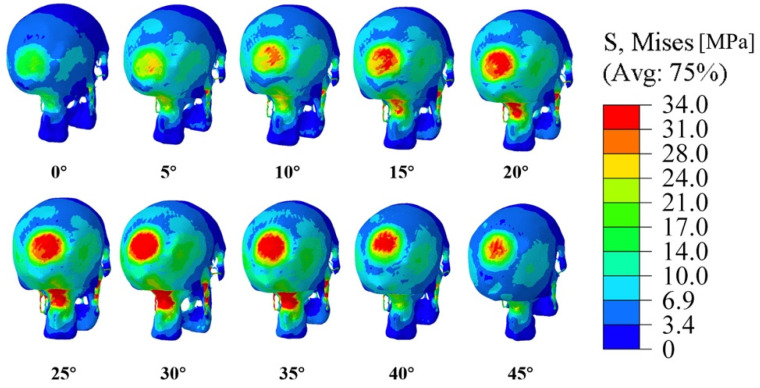
Stress nephograms generated from simulations involving different contact angles at 3.5 m/s.

Next, we focus on the case with the impact speed of 3.5 m/s, which would cause the most severe damage on the head model. Figure 7 shows the stress nephograms generated from simulations at that speed. From those nephograms, apparent differences in head damage caused by the head–ground impacts with different contact angles can be found. In addition to the skull fracture, moderate to serious injuries also occur at the junction between the skull and the spine. Again, that figure shows that the threshold value of 34.47 MPa is first reached and exceeded at the contact angle of 30°.

**Figure 8:**
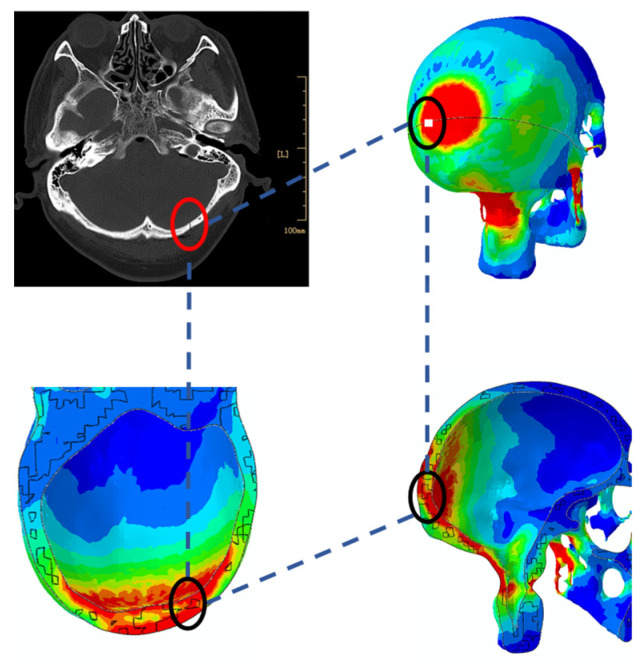
Comparison between simulation results with the CT image.

As shown in Figure 8, when the contact angle between the head and the ground is 30°, the stress at the detection position is the largest. Furthermore, at 3.5 m/s, the stress at the detection position is 37.84 MPa, which exceeds the skull fracture threshold of 34.47 MPa given in Table 4. Therefore, we decided that the lowest speed that would cause the damage on the head would be 3.5 m/s and at that speed, the contact angle between the head of the injured person and the ground is around 30°. As the impact speed increases, the maximum stress obtained at other impact angles will also reach the threshold value of 34.47 MPa, the probability of serious injury and even death will duly increase.

## 4. Validation

**Figure 9:**
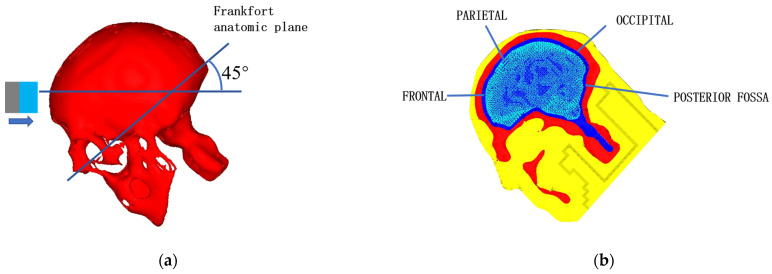
FE model for Nahum’s #37 test [35]: (**a**) the model, (**b**) the regions where the intracranial pressures were calculated.

After the analysis, the developed model was used to repeat the famous cadaver head impact experiment (test #37) conducted by Nahum [35] and the simulation results were compared with Nahum’s data for validation and verification. In Nahum’s test, a 5.59 kg cylinder object impacted the frontal part of a head at an initial speed of 6.3 m/s. Figure 9a shows the FE model of that test setup, in which the boudary conditions were defined to reflect the scenario of the original test. The material of the impactor was assumed to be rigid, with Young’s modulus of 6 MPa and Poison’s ratio of 0.16. In the post-processing face, intracranial pressures on the frontal area, parietal area, occipital area, and posterio fossa of the head were plotted and compared with the results obtained from Nahum’s test.

**Figure 10:**
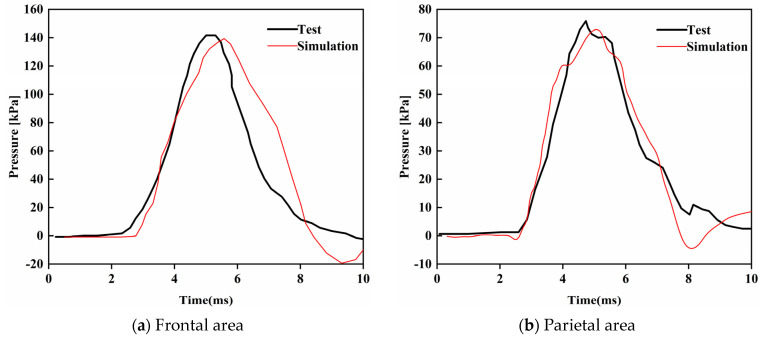

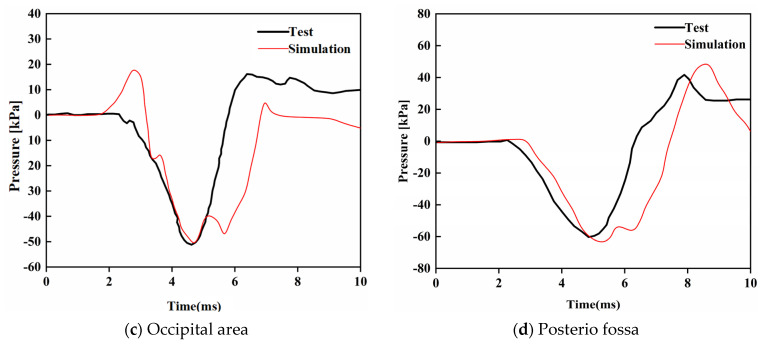
Comparison between computational and experimental results.

In previous simulations, the calculated peak pressures usually occurred sooner than the measured peak pressures because the scalp and other tissues were ignored in the FE models, and therefore their buffering effect was not taken into consideration in the past simulations. To improve the biofidelity of FE head models and accuracy of simulations, the scalp was carefully modeled and integrated into the present head model. Figure 10a–d compares the simulation results and experimental data obtained from the four areas as specified in Figure 9b. From those figures, it can be found that the simulation results are in good agreement with the experimental data, expecially for the intracranial pressures on the frontal and parietal area. The present model and the modeling approach are therefore verified.

## 5. Discussion

This study only discusses the effects of impact speed and contact angles on the human head injury and only the threshold for skull fracture is used. In fact, there are other factors such as weather, road conditions, and human factors that contribute to a traffic crash and normally more than one criterion should be used to evaluate the human injury. Moreover, only direct impact injury between the head and the ground is considered in this study because the main goal of this study is to establish a reliable computational modeling framework in support of the investigation of head injuries caused by traffic accidents. In the next phase, more comprehensive models will be established based on the present model and the proposed modeling approach will be followed, which will take different factors into account and implement more human injury criteria so as to achieve more faithful reconstructions of the traffic crashes and better predictions of traumatic head injury caused by the head–ground impacts incurred in the accidents. Actual head injuries including rotational shear stress and burst injury, as well as secondary injuries such as hypoxia, hypotention, brain edema, and epilepsy will be further analyzed using the advanced models. The outcomes of future research will help physiatrists and nurses address clinically significant problems.

## 6. Conclusions

The present study uses Hypermesh and ABAQUS/Explicit to model and simulate 60 collisions between a human head and the ground which involve different contact angles and impact speeds. On the basis of the numerical results and comparison of those results with the experimental data, the minimum speed that would lead to skull fracture and the corresponding contact angle were determined. The following conclusions can be drawn from this study:

(1) At the same impact speed, the maximum peak von Mises stress is reached when the contact angle is 30°.
(2) The maximum peak stress increases as the impact speed increases and when the impact speed is 3.5 m/s at the contact angle of 30°, the peak stress generated at the impact exceeds the threshold value and would cause the skull to fracture.
(3) FE methods are a powerful tool for reconstructing scenes of traffic crashes and reproducing impact processes in a time- and cost-effective manner. The combination of the FE methods and experimental data can reveal valuable insights into traffic crashes and assist forensic analysts in identifying the responsibilities of involved parties and revealing the courses of accidents.

The developed model and modeling approach can be applied to predict and reconstruct head/skull injuries in traffic accidents, which cannot be reconstructed in reality. Simulation results obtained from the developed model can be used to augment experimental datasets of limited volume to provide researchers with wider and more detailed data in support of their investigation of head/skull injuries and optimization for pedestrian head protection.

## Figures and Tables

**Table 1:** The total element numbers for different parts of the human head.

Component	Skull	Brain	Cerebrospinal Fluid	Scalp and Skin
No. of elements	229,176	52,636	113,083	242,885

**Table 2:** Elastic material properties for the head model and the ground (the data come from [8,10]).

Component	Density (kg/m^3^)	Young’s Modulus	Poisson’s Ratio
Skull	2.0 × 10^3^	20.1 GPa	0.28
CSF	1.0 × 10^3^	1.20 MPa	0.49
Scalp, Skin, etc.	1.2 × 10^3^	16.70 MPa	0.42
Ground	7.8 × 10^3^	210 GPa	0.30

**Table 3:** Viscoelastic material properties for the head model and the ground (the data come from [8,10]).

Component	Density (kg/m^3^)	Young’s Modulus	Short-Time Shear Modulus	Long-Time Shear Modulus	Decay Constant
Brain	1.04 × 10^3^	2.19 GPa	0.528 MPa	0.168 MPa	80

**Table 4:** Head injury threshold criteria.

Form of Injury	Parameters	Threshold
Brain damage	ICP	Damage (>235 KPa) Small or non-injury (<173 KPa)
	Cut stress	Severe injuries (11~16.5 KPa).
	Von Mises stress	Bruising (7~8.6 KPa). Mild traumatic brain injury (>20 KPa) Concussion (15~20 KPa) Shaft rope damage (>26 KPa)
Skull fracture	Von Mises stress	Skull fracture (>34.47 MPa)

## Data Availability

Not applicable.
